# Interaction with SMS text-reminders correlate with improved medication adherence and readmission rates for congestive heart failure patients: A retrospective cohort study

**DOI:** 10.1371/journal.pdig.0001157

**Published:** 2025-12-31

**Authors:** Ben Long, Brian Davis, Rebekah McPheters, Steven Burton, Nabeel Hamoud, Dan Garmat, Suzanne Catalfomo, Fei Li, Ying Zhou, Yan L. Zhuang, Colin A. Banas, Weston W. Blakeslee

**Affiliations:** 1 Department of Internal Medicine, Magnolia Regional Health Center, Corinth, Mississippi, United States of America; 2 Department of Applied Clinical Research, DrFirst, Rockville, Maryland, United States of America; ZEW - Leibniz Centre for European Economic Research: ZEW - Leibniz-Zentrum fur Europaische Wirtschaftsforschung GmbH Mannheim, GERMANY

## Abstract

Short message service text reminders (SMS nudges) aimed to help vulnerable patient populations remember to fill their prescriptions are becoming more common but accurately measuring their effects on improving prescription fill and readmission rates remains challenging. Patients who presented to the emergency department (ED) with a primary diagnosis of congestive heart failure (CHF) were included in the study. We conducted a retrospective cohort study of CHF patients who did and did not interact with SMS nudges, then matched patients who were prescribed medications at any point in their hospital visit with records of subsequent prescription fills. Patients that interacted with SMS nudges had 19% higher odds of filling prescriptions overall (1.19 OR (95% CI: 1.15 – 1.24), p < 0.001) and 6% lower odds of being readmitted to the hospital (0.94 (95% CI: 0.9 – 0.99), p = 0.009) than patients who did not interact with SMS nudges. Interactive SMS nudges via a novel tool may improve prescription fill rates across multiple groups of CHF patients, and contribute to a reduction in readmissions.

## Author summary

When patients leave the hospital with new prescriptions, many never fill them. A problem that can lead to worsening symptoms and costly hospital readmissions. We wanted to investigate if personalized text message reminders could help patients remember to pick up their medications after being hospitalized for heart failure. We studied over 1,200 heart failure patients at a rural hospital who received text messages reminding them about their prescriptions. These weren’t just simple reminders. Patients could click a link to see which medications were waiting, watch educational videos, and access coupons to reduce costs. Our findings suggest that patients who engaged with these text reminders were more likely to pick up their prescriptions and less likely to return to the hospital within 30 days. The effect was strongest among patients who had been readmitted. Those who clicked on the reminder increased their prescription fill rates by nearly 10 percent. Our results suggest personalized SMS-text reminders may be a simple, scalable tool to help vulnerable patients manage their medications. This matters because even small improvements in medication adherence could prevent emergency room visits and hospitalizations, ultimately improving patients’ quality of life.

## Introduction

In the United States (US), between 20–30% of all prescriptions are abandoned [1–3] producing an additional $100–290 billion dollars of non-value-added burden [4]. Medications that are abandoned are often prescribed for chronic conditions to reduce adverse events that lead to emergency room visits and costly hospitalizations [5]. Of these predominant chronic conditions, congestive heart failure (CHF) is a leading discharge diagnosis [6]. Medication non-adherence among adult patients with CHF are concerning [7–9], with most investigators citing rates of 40% to 60% [6]. Not only are nearly 1 in every 4 CHF patients readmitted within 30 days of discharge, but rates also increase to 50% within 6 months of discharge in the US [10,11]. High nonadherence and readmission rates underscore the need of better care for this patient group.

Many strategies are employed to improve medication adherence for CHF patients [12]. These strategies target multifactorial aspects of CHF, including but not limited to: the clinical complexity of CHF diagnosis, emphasis on medication management, and improving quality of life [13,14]. One of the positive outcomes that emerged from the COVID-19 pandemic was the overall increase in digital patient engagement strategies, such as: telemedicine, patient portal adoption, and SMS text messaging for a variety of healthcare related tasks. According to an AMA survey, approximately 85% of physicians currently engage with patients through telemedicine, while preceding rates of virtual care in 2016 were only 14% [16]. Additionally, 70% of these providers expect to continue utilizing telemedicine in the post-COVID era [15]. Inspiring the use of patient portals prior to the pandemic posed a significant challenge for health system informaticists. However, since the onset of COVID-19, there has been a noticeable increase in patient portal registration and usage. Healthcare systems nationwide have increasingly leveraged these portals to expand access to healthcare services and foster greater patient engagement. Despite the growing reliance on asynchronous communication through patient portals, significant disparities in usage persist across various demographic subgroups [16–20]. These subgroups include persons 70 years and older, women, racial and ethnic minorities, and those with limited health literacy [21].

As an additional effort, text messages have been supplemented as another communication channel, mainly due to their public accessibility and efficiency. Patient engagement rates with text messages also appear to be an effective manner to address healthcare needs, with a recent study reporting 90% engagement with text messages from their healthcare organization, and over 90% rates in appreciation of text messages, better interconnection with patient’s care teams, and identifying the needs for telephonic assistance from their provider’s office [19].

Interventions like short message service texts (SMS nudges) have shown to be successful in managing diabetes, weight loss, diet, medication adherence, and other clinically relevant issues [22]. Additionally, personalized messages may have even greater effects than messages that are not specifically tailored to patients [22]. With the near ubiquitous availability of the US public to receive SMS text messages [23], SMS nudges were utilized to improve medication management of CHF patients in our health system. The goal of this analysis was to assess the effectiveness of interactive SMS nudges on prescription fill rates and readmission odds for CHF patients to assess the clinical applicability of the digital health tool, and to inform a future prospective rigorously controlled study.

## Methods

### Setting, intervention, participants, and study design

This study was conducted at a rural hospital in the southeastern United States. Patients were sent a text message 5 minutes after an e-prescription was submitted in the hospital’s Electronic Health Record (EHR). Individual patient consent was inherited and honored from previous recording of patient communication preference at the hospital. The patient’s doctor could opt their patient out of SMS nudges at the encounter level, and patients could opt out of SMS nudges via the SMS message itself or after authenticating their identity.

**Fig 1:**
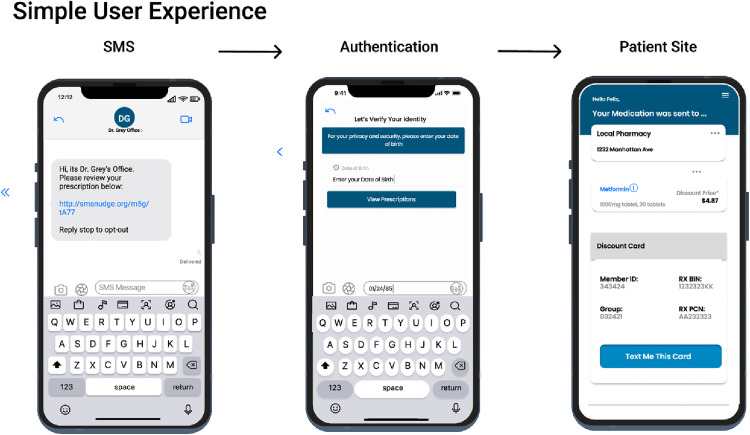
Patient end-user experience for SMS-text reminders. Simple workflow of the SMS user experience. A personalized message is presented to the patient with a link to a micro-site. The patient then enters their birthdate to authenticate their identity. Finally, a patient receives details and education about their medications.

**Fig 2:**
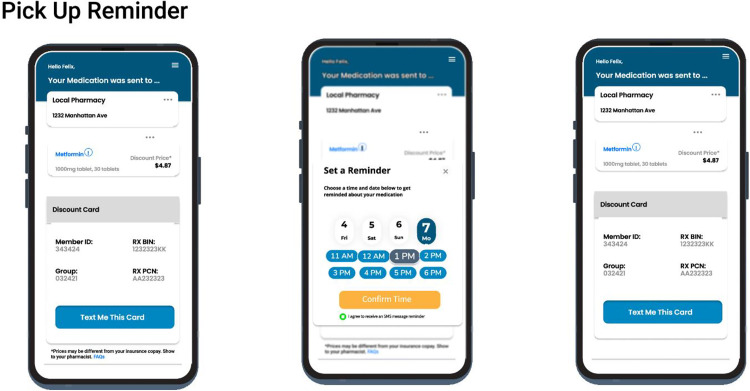
Patient-initiated medication pick-up reminder. Medication pick-up reminder workflow for patients. Navigation to the reminder scheduler stems from the main landing page of the micro-site and walks the patient through steps to schedule a medication pick- up reminder. Consent to receive a text reminder is provided, and the patient navigates back to the primary landing page.

The SMS nudge intervention is interactive and includes a link that is customized to each patient that receives it. The initial SMS nudge messaging includes wording identifying the patient’s prescriber. Upon clicking the link there is content that shows prescription destination information, educational content (both written and video) as well as, when applicable, coupon and co-pay assistance cards with pricing information embedded in the interaction (Figs 1 and 2), providing an interactive patient experience when reminded to pick up their prescriptions.

Patients were grouped in to three categories: Non-Readmitted (patient had an initial inpatient visit with a primary diagnosis of CHF and no subsequent Emergency Department (ED) visits), Return Visit to ED (patient has a return visit to the ED within 30 days of visit with primary diagnosis of CHF, but was stabilized and discharged from the ED), and Readmitted to Inpatient (IP) (patient has a return visit to the ED within 30 days of visit with primary diagnosis of CHF and was deemed to need a repeat inpatient stay.)

### Study eligibility criteria

Patient demographics were recorded for individuals that presented as an inpatient with a primary diagnosis of CHF (International Classification of Diseases, version 10, Clinical Modification (ICD-10-CM) of CHF (I09.**, I26.**-I28.**, I50.**, Z86.**). Patients were eligible for inclusion to receive SMS nudges to fill their prescriptions if: they are not a minor, own a phone that is SMS-capable with correct configuration, and meeting minimum payload data standards (such as a smartphone that can render websites, not a simple flip phone), have a record of their phone number in the hospital’s EHR, and received an electronic prescription from a provider from the hospital during the study period.

### Patient matching

**Fig 3:**
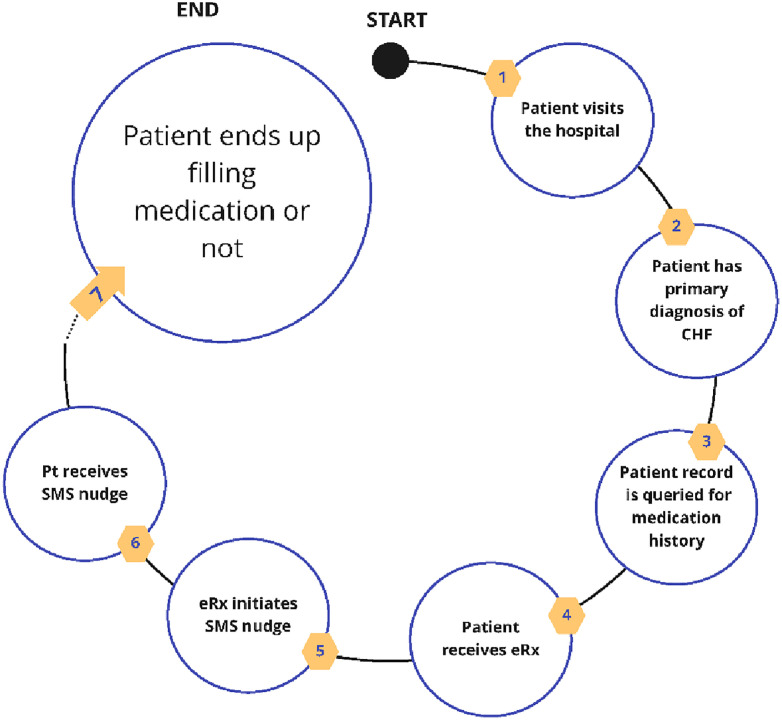
Workflow of text message reminder intervention for prescription fills. Schematic representation of the workflow used in the study to assess the impact of text message reminders on prescription fills. The diagram outlines the sequence from participant enrollment, sending of text message reminders, to tracking prescription fill rates. This workflow highlights the intervention steps, and the data collection points throughout the study.

Patients that were found in the demographics dataset that was generated from the hospital’s EHR were matched to a separate database along four demographic variables first name, last name, date of birth, and zip code of primary residence. Case-insensitive, exact matching was used to conservatively match patients. This allowed patients to be matched to a unique patient identifier that is created after the first electronic prescription (eRx) for that patient. Next, all eRx transmissions were queried for each patient, as well as subsequent medication fills, and SMS text metrics and interactions, defined as clicks in the SMS nudge patient experience (Figs 1 and 2). Patient’s medication history was queried using five demographic variables: first name, last name, date of birth, gender, and zip code. All pharmacy claims and fill records were mapped to the unique patient identifier to determine if a prescription fill occurred (Fig 3).

### Defining prescription abandonment

We define prescription abandonment as the presence of an eRx that has no record of a fill in the baseline and intervention period. Data for prescription abandonment was taken from May 26^th^, 2016 (the date in which demographic data was initially recorded) to March 27^th^, 2022 (the end of the intervention period). The date range of the intervention was December 16^th^, 2019 (the date of the first successfully delivered SMS nudge) to March 27^th^, 2022.

### Data analysis

We employed logistic regression modeling to estimate the associations between SMS nudge engagement and clinical outcomes. From the coefficients of these models, we were able to estimate odd ratios through standard approaches (taking the antilog of the regression coefficients). Models were adjusted for maintenance drug status (binary variable, determined by the study’s clinicians) and patient age (in years). All analyses were carried out using python 3.9.11 [24]. For logistic regression model we employed the `statsmodels` package [25] and for data transformations we used the `pandas` package [26].

### Ethics statement

This retrospective analysis used de-identified patient data collected during routine clinical care. As a result, the Magnolia Regional Health Care Ethics Committee waived ethical approval due to the minimal risk and non-interventional nature of the study. Data handling complied with the Declaration of Helsinki (2024) standards for privacy and patient confidentiality throughout the analysis.

## Results

### Demographics

Table 1 depicts our patient demographics. Over the study period, 1,276 patients met the inclusion criteria for the baseline analysis. Minimal criteria included being admitted to the hospital with a primary diagnosis of CHF and receiving eRxs during the hospital visit. The median age of these patients was 76 years old [min age: 24 – max age: 105]. Most patients were male (52.3%). Insurance coverage varied between Medicare, Medicaid, Dual-Eligible, Veterans Affairs Health Benefits (VA) and commercial insurance, with Medicare being the predominant insurer at 73.5%. The average length of stay (LOS) was 3.69 days (95% CI = 3.51 – 3.88). The average readmission rate during the study period was 25.6% (95% CI = 23.2% - 28.0%). Dispositions varied but were most common across these predominant conditions: Home Self Care (58.9%), Home Health Care (21.1%), and Transferred (15%). The most common readmission diagnoses were cardiovascular conditions (7.9%) and respiratory conditions (2%).

**Table 1:** CHF patient demographics (CHF scorecard).

Variable	Value [n(%)]
Number of Patients	1276
Age	
18-24	1 (0.1)
25-34	2 (0.2)
35-44	26 (2.0)
45-54	101 (7.9)
55-64	179 (14.0)
65-74	288 (22.6)
75-84	383 (30.0)
85-94	248 (19.4)
95-105	48 (3.8)
Gender	
Male	667 (52.3)
Female	607 (47.6)
Other or Missing	2 (0.2)
Primary Insurance	
Medicare Advantage	68 (5.3)
Medicare Only	938 (73.5)
Medicaid Only	18 (1.4)
Commercial Insurance	159 (12.5)
Veterans Insurance	23 (1.8)
Self-Pay Insurance	63 (4.9)
Other	7 (0.5)
Length of Stay (days)	
0	172 (13.5)
1	79 (6.2)
2	263 (20.6)
3	230 (18.0)
4	164 (12.9)
5	128 (10.0)
6 - 10	192 (15.0)
11 - 20	44 (3.4)
21 - 30	3 (0.2)
31-50	1 (0.1)
Disposition	
Home Health Care	269 (21.1)
Home Self Care	751 (58.9)
Unskilled Assisted Living	3 (0.2)
Transferred	192 (15.0)
Hospice	26 (2.0)
Expired	27 (2.1)
Other	8 (0.6)
Readmit Primary Diagnosis	
Cardiovascular Condition	101 (7.9)
Heart Failure	15 (1.2)
Sepsis	12 (0.9)
Respiratory Conditions	26 (2.0)
Liver Failure	2 (0.2)
Kidney Failure	13 (1.0)
Other	54 (4.2)
Missing	1053 (82.5)

This table represents the demographics of patients included in the CHF scorecard analysis.

### Descriptive statistics

In Table 2, we present the available eRx, medication fill records, and successful interactive SMS nudge deliveries across our patients by group. Patients with a primary diagnosis of CHF were grouped by Not Readmitted (949), Return visit to ED (104), and Readmitted to IP (223). The included patients all received at least one prescription during their hospital visit. Medication fills were tracked following eRxs, and the percentage of unique patients with confirmed medication fills were 80.5% for Not Readmitted, 87.5% for Return visit to ED, and 82.1% for Readmitted to IP. Interactive SMS nudges were sent to all eligible CHF patients that received an eRx, with 68.9% of patients in the Not Readmitted group, 72.1% for Return visit to ED, and 57.4% for Readmitted to IP group receiving SMS nudges.

**Table 2:** Descriptive statistics for CHF patients (patient totals, SMS text engagement rates).

2A. eRx, Medication History Records, and SMS Text at at Patient Level					
	eRx (n)	Medication History (n)	Medication History/ eRx (%)	SMS Text (n)	SMS Text/ eRx (%)
**Not Readmitted**	949	764	80.5%	654	68.9%
**Return Visit to ER**	104	91	87.5%	75	72.1%
**Readmitted to IP**	223	183	82.1%	128	57.4%
**Total**	1276	1038	81.3%	857	67.2%
**2B.** Messages and Clicks at Patient Level (CHF Patient Population)					
	**Messages Sent (n)**		**Ever Clicked (n)**	**Ever Clicked (%)**	
**Not readmitted**	654		599	91.6%	
**Return Visit to ER**	75		68	90.7%	
**Readmitted to IP**	128		124	96.9%	
**Total**	857		791	92.3%	
**2C.** Messages and Clicks at eRx Level					
	**Messages Sent**		**Ever Clicked**	**Ever Click (%)**	
**Not Readmitted**	23710		15194	64.1%	
**Return Visit to ER**	2540		1514	59.6%	
**Readmitted to IP**	5128		3258	63.5%	
**Total**	31378		19966	63.6%	

All numbers represent unique patients in each group.

Additionally in Table 2, we present the SMS nudge metrics at the patient and medication levels across the three patient groups. Patient behavior was measured through interaction with the SMS nudge at the patient level as well as the individual message level. The percentage of patients that clicked the initial link in the SMS nudges at least once was over 90% for all three patient groups, with the largest proportion of patients interacting at 96.9% in the Readmitted to IP group. Patients were permitted to opt out of receiving SMS nudges after the first message. Only 7.7% of patients that successfully received SMS nudges (66/857) decided to opt out of the program. For all messages that patients received, around 60% were clicked across the patient groups. 64.1% of messages sent to Not Readmitted patients were clicked, followed by 59.6% of message clicks in the Return Visit to ER group, and 63.5% of message clicks in the Readmitted to IP group.

### SMS nudge effect on medication fill rates

The average medication fill rates across all maintenance medications and all patient groups was 60.4% (S1 Table A). Medication fill rates of CHF specific medications was 60.6% (S1 Table B). Patient behavior in click rates and prescription fill rates were recorded in Table 3 across all patient groups. Patients who clicked the SMS nudge in the Not Readmitted group increased their raw prescription fill rates by 3.2% (58.2% => 61.4%), which also increased the odds of filling a medication by 15% (OR: 1.15, 95% CI: 1.1 – 1.2, p < 0.001). In the Return Visit to ER group, there was a slight decrease in fill rates after interacting with SMS nudges (59.2% => 58.6%), but no statistically significant difference in the odds of filling a medication (0.97 (95% CI: 0.86-1.11), p = .692).The Readmitted to IP group saw the largest increase, both in fill rates (50.3% => 60.2%) and with a statistically significant increase in the odds of filling a medication after clicking the SMS nudge of 52% (OR: 1.52, 95% CI: 1.37 – 1.69, p < 0.001). We adjusted the raw analysis to control for: age, maintenance medications, and CHF medications to reduce variability. The overall effect of clicking SMS nudges on the odds of filling all medications across all groups was 19% (OR: 1.19, 95% CI: 1.15 – 1.24, p < 0.001).

**Table 3:** SMS text effect on prescription abandonment and readmission odds for CHF patients.

3A. SMS Text Clicks vs Filling Status (All Medications)					
	Clicked Flag		% of not Filled	% of Filled	Total Rx
**Not Readmitted**	**Not Clicked**		41.8	58.2	15639
	**Clicked**		38.6	61.4	16273
**Return Visit to ER**	**Not Clicked**		40.8	59.2	1675
	**Clicked**		41.4	58.6	2154
**Readmitted to IP**	**Not Clicked**		49.7	50.3	3339
	**Clicked**		39.8	60.2	2871
**Total**			41.0	59.0	41951
**3B.** Raw SMS Text-Filling Odds Ratio:					
				**Odds ratio (with 95% CI)**	
**Not readmitted**				1.14 (95% CI: 1.09-1.19), p < 0.001	
**Return Visit to ER**				0.97 (95% CI: 0.86-1.11), p = .692	
**Readmitted to IP**				1.49 (95% CI: 1.35-1.65), p < 0.001	
**Overall**				1.18 (95% CI: 1.13-1.22), p < 0.001	
**3C**. Adjusted SMS Text-Filling Odds Ratio					
				**Odds ratios (with 95% CI)**	
**Not readmitted**				1.15 (95% CI: 1.1-1.2), p < 0.001	
**Return Visit to ER**				1.02 (95% CI: 0.89-1.16), p = 0.772	
**Readmitted to IP**				1.52 (95% CI: 1.37–1.69), p < 0.001	
**Overall**				1.19 (95% CI: 1.15-1.24), p < 0.001	
**3D.** SMS Text Clicks and Readmittance Associations (Total Population)					
**Click Flag**		**Not Readmitted**		**Return Visit to ER and Readmitted to IP**	**Total Rx**
**Not Clicked**		15639		5014	20653
**Clicked**		16273		5025	21298
**Total**		31912		10039	41951
Raw odds ratio: 0.96 (95% CI: 0.92-1.01), p = 0.101 Adjusted odds ratio: 0.94 (95% CI: 0.9-0.99), p = 0.009					
**3E.** SMS Text Clicks and Readmittance Associations (Only Among CHF Patients)					
		**Not Readmitted**		**Return Visit to ER and Readmitted to IP**	**Total Rx**
**Not Clicked**		4922		1509	6431
**Clicked**		4873		1464	6337
**Total**		9795		2973	12768
Raw odds ratio: 0.98 (95% CI: 0.9-1.06), p = 0.628 Adjusted odds ratio: 0.96 (95% CI: 0.89-1.04), p = 0.35					
**3F.** Readmittance Associations (Total Population)					
				**Odds Ratio (95% CI)**	
**Raw Odds Ratio**				0.85 (95% CI: 0.58-1.27), p = 0.434	
**Adjusted Odds Ratio**				0.86 (95% CI: 0.58-1.29), p = 0.469	

This table represents the odd ratios of medication fulfillment and readmittance associated with SMS clicks.

### SMS nudge effect on readmissions

We investigated whether interaction with SMS nudges had any correlative effect on the odds of a readmission in our CHF patient population. In Table 3, we observed a reduction in the odds of a readmission if a patient interacted with SMS nudges of 6% after controlling for age and maintenance drug status at the medication level (OR: 0.94, 95% CI: 0.9 – 0.99, p = 0.009). When we assessed whether interaction with SMS nudges correlated with the odds of a readmission at the patient level, the odds decreased lower than 6% (14%), but due to the variability in the confidence interval, the value was not statistically significant (OR: 0.86, 95% CI: 0.58 – 1.29, p = 0.469). Patients from the Return Visit to ED and Readmitted to IP were grouped together in this analysis.

## Discussion

We believe it is necessary in this analysis to establish a robust baseline of demographics and prescription fill rates, due to the ever-increasing efforts of improving clinical outcomes in CHF patient populations and the scarcity of studies that rigorously measure fill rates specifically in CHF patients. Readmission rates, average age, LOS and other demographic metrics for CHF patients in our community were on par for national averages when it comes to CHF [10,11,13,14]. The percentage of first fills across maintenance medications and CHF-specific medications was around 40% (S1 Table A), which is higher than reported national averages across all patients at 20–30% [1,3]. This is an important consideration with the clinical literature reporting that reduction in prescription fill rates can lead to worsening symptom management for chronic conditions [27–31]. We included a longer time frame of data for our baseline metrics because the more data points that are available, the more accurate the state of the baseline, and these results will be useful in subsequent meta-analyses.

We measured the effect of interactive SMS nudges on prescription fill rates across different groups of particularly vulnerable CHF patients and establishing plausibility for 30-day readmission reduction in these patients. The percentage of CHF patients with records of at least one fill were above 80% in all groups and highest in the Return Visit to ED group at 87.5% (Table 2A). This is slightly higher than the percentage of all patients with at least one fill in the entire health system at 81% (data not shown). The outcomes for interacting with SMS texts at the patient level (92.3%), eRx level (59.6%), and the opt-out rate was 7.7% in our study (n = 857, Table 2C) were all considered successful measures by the study authors. The opt-out rate in the recent NUDGE feasibility study was 14% (n = 400) [32], though the n of patients was smaller.

The most significant effect of the interactive SMS nudges occurred in the Readmit to IP group, even though only 57.4% of this population was reached by SMS nudges (Table 2A). In this group, click rates for SMS nudges was the highest (96.9%, Table 2B), raw fill rates increased by the highest margin (9.9%, Table 3A), and odds of clicking on SMS nudges to corresponding fill rates was the highest (52%, Table 3C). The most perplexing patient group were patients that had a Return Visit to ER within 30 days but were not readmitted to an inpatient setting. This group did not react as well to SMS text reminders. With overall click rates being ~4% less in the Return Visit to ER group than the other two groups (Table 2C); raw fill rates decreasing 0.6% (Table 3A); and odds of filling (1.02, p = 0.772, Table 3C) were all lower in the Return Visit to ER group. Perhaps patients that stayed healthy enough to not be readmitted and patients that had more severe symptoms re-visiting the hospital were overall more motivated to fill their prescriptions regularly.

We observed statistically significant associations between patients who interacted with SMS nudges and higher medication fill rates in the Not Readmitted (15% OR, p<0.001) and Readmitted to IP (52% OR, p<0.001) (Table 3C), but these associations may reflect selection bias rather than causal effects from patients who interact with digital health tools versus patients who do not. We also wanted to investigate if engagement with these reminders had an effect reducing the odds of getting readmitted. Based on our analysis, patients who clicked on the interactive SMS nudge observed reduced odds of readmissions at 6% (p = 0.009 Table 3D), suggesting that improved prescription fill rates from interactive SMS nudges may play a role in reducing readmission odds. Though confounding variables such as patient motivation, social support, and symptom severity for our CHF population cannot be ruled out.

An interesting observation at the medication level was the total prescription abandonment level being ~10% higher for Entresto (49.1%) than the average of all maintenance meds (39.6%), and much higher than ACE inhibitors (41.2%) and beta- blockers (38.9%) (S1 Table A). There is very likely a cost component to this observation with Entresto typically costing hundreds of dollars a month versus generic ACE inhibitors and beta-blockers costing under $10 a month. Incorporating prescription price transparency’s effect may be additive to the effect of interactive SMS nudges.

From the existing evidence in the literature [23] and the results of this study, we believe personalization of the SMS message is very important and suggest that all follow-on studies include this level of patient relatability in their methods. Tailoring text messages to be representative of their familiar medical care may significantly improve the patient experience when filling discharge prescriptions. We also suggest that electronic prescription data, medication fill data, SMS text delivery and interaction data, and robust patient matching are required for accurate assessments of prescription abandonment rates and measuring interventions aimed at improving medication adherence.

There is good evidence that support the claim that filling prescriptions more regularly improves clinical outcomes [29–31]. In our retrospective analysis, we observed that interaction with SMS nudges was associated with higher prescription fill rates and lower 30-day readmissions. However, the observational design of our study precludes causal inference. A future randomized controlled trial will be necessary to show causality of SMS nudges or whether these effects reflect other confounding variables. Follow on efforts will measure if our method has an impact on other secondary outcomes, such as: proportion of days covered (PDC), blood pressure, and cardiovascular clinical events. We believe that Interactive SMS nudges help enrich the patient experience in filling prescriptions, when in the past, patients were on their own when it came to obtaining their medications. Currently, a large-scale multi- institution study that is measuring the effectiveness of SMS delivered messages to improve adherence in cardiovascular disease are underway and it will be interesting to see those results [22,31].

Our study does have several limitations. The authors realize that interaction with SMS nudges is only a part of the entire picture for improving prescription fill and readmission rates. Even receiving the SMS nudge may influence fill rates without clicking on the nudge. We acknowledge that the primary focus of this study was measured on the success rate of discharge prescriptions. Ambulatory prescription fills were outside the scope of our study, though would be interesting to investigate in the future. Direct patient education, verbal reminders, pharmacist support, automated telephone communications, digital timer caps, and financial incentives are other methods to improve medication adherence [22] and were not considered in this study. A potential disadvantage of our analysis was excluding patients who may not have access to a smartphone due to low socioeconomic status. Though smartphone use is prevalent in the United States, this is a limitation worth considering. Efforts were made to simplify the analysis to find meaningful signals with these limitations in mind. Some local dispensing pharmacies do not share their pharmacy fill data due to the differing policies of local and independent pharmacies with regards to sharing prescription data. While some pharmacies have “opt-out” data sharing agreements, most have “opt-in” requirements. As such, a few pharmacies are not able to share their data with a third-party data aggregator. An ancillary analysis from our external data source vendor indicates that over 92% of all patients that were queried for medication history were found to have prescription fills; thus, a small portion of patients may be missing specific pharmacy claims.

Though there was a statistically significant reduction in the odds of CHF patient readmissions at the population level when controlling for maintenance medications, a wide confidence interval at the patient level showed a trend in reduced readmissions at the unique patient level but was not statistically significant. This may become clearer with a larger patient sample size in future studies.

## Conclusions

Though there are many IT tools and clinical programs to improve medication adherence, it remains an enormous problem that, if solved, would prevent many negative downstream effects in the disease management of patients. Our study follows a prescription through the patient’s journey of care and measures the effect of an interactive SMS nudge intervention on prescription abandonment, medication adherence, and the likelihood of getting readmitted to the hospital after being reminded to pick up prescription medications. CHF patients that are readmitted abandon the first fill of their prescriptions more often; engaged with our secure text message reminder service less often; and are more likely to be readmitted to the hospital if they do not engage with the secure text message reminder service. However, if a patient that was readmitted did engage with the secure text message, the percentage increase in fill rates was higher than patients that were not readmitted. Our findings provide preliminary evidence suggesting that interactive SMS nudges may improve medication adherence and clinical outcomes in CHF patients. A randomized controlled trial is warranted to determine if SMS nudges causally improve medication fill rates and reduce readmissions, or whether our observations reflect patient selection effects.

## Supporting information

S1 Table This supporting table represents total Rx fill counts, Rx fill %, and Rx Abandonment % broken out by all maintenance medications and select drug subclasses for the study cohorts. (DOCX)

S2 Table This supporting table shows the included drug classes in our analyses for treatment of Congestive Heart Failure. (DOCX)
