# Supplementary material for: Interaction with SMS text-reminders correlate with improved medication adherence and readmission rates for congestive heart failure patients: A retrospective cohort study
Source: PLOS Digit Health. 2025 Dec 31;4(12):e0001157. doi: 10.1371/journal.pdig.0001157 (PMC12755802; doi:10.1371/journal.pdig.0001157)
Supplement: S1 Table — (DOCX) [file pdig.0001157.s001.docx]

### Supporting Information 1 Table. Additional Prescription Abandonment Statistics by Drug Class

| **S1 Table A.** Prescription Abandonment for all Maintenance Meds | | | | | |
| --- | --- | --- | --- | --- | --- |
|  | **Rx Not Filled** | **Rx Filled** | **Total Rx** | **Rx Filled %** | **Rx Abandonment %** |
| **Not Readmitted** | 9069 | 14306 | 23375 | 61.2% | 38.8% |
| **Readmitted to ER** | 1024 | 1554 | 2578 | 60.3% | 39.7% |
| **Readmitted to ER and IP** | 1925 | 2445 | 4370 | 55.9% | 44.1% |
| **Total** | 12018 | 18305 | 30323 | 60.4% | 39.6% |
| Note: Abandonment in both readmitted groups: 42.4% | | | | | |
| **S1 Table B.** Prescription Abandonment of all CHF Meds | | | | | |
|  | **Rx Not Filled** | **Rx Filled** | **Total Rx** | **Rx Filled %** | **Rx Abandonment %** |
| **Not Readmitted** | 3809 | 5986 | 9795 | 61.1% | 38.9% |
| **Readmitted to ER** | 400 | 654 | 1054 | 62.0% | 38.0% |
| **Readmitted to ER and IP** | 823 | 1096 | 1919 | 57.1% | 42.9% |
| **Total** | 5032 | 7736 | 12768 | 60.6% | 39.4% |
| Note: Abandonment in both readmitted groups: 41.1% | | | | | |
| **S1 Table C.** Prescription Abandonment of ARNi | | | | | |
|  | **Rx Not Filled** | **Rx Filled** | **Total Rx** | **Rx Filled %** | **Rx Abandonment %** |
| **Not Readmitted** | 125 | 136 | 261 | 52.1% | 47.9% |
| **Readmitted to ER** | 18 | 11 | 29 | 37.9% | 62.1% |
| **Readmitted to ER and IP** | 26 | 28 | 54 | 51.9% | 48.1% |
| **Total** | 169 | 175 | 344 | 50.9% | 49.1% |
| Note: Abandonment in both readmitted groups: 53.0% | | | | | |
| **S1 Table D.** Prescription Abandonment of ACE inhibitors | | | | | |
|  | **Rx not filled** | **Rx filled** | **Total Rx** | **Rx Filled %** | **Rx Abandonment %** |
| **Not Readmitted** | 368 | 538 | 906 | 59.4 | 40.6% |
| **Readmitted to ER** | 25 | 54 | 79 | 68.4% | 31.6% |
| **Readmitted to ER and IP** | 65 | 62 | 127 | 48.8% | 51.2% |
| **Total** | 458 | 654 | 1112 | 58.8% | 41.2% |
| Note: Abandonment in both readmitted groups: 43.7% | | | | | |
| **S1 Table E.** Prescription Abandonment of Beta Blockers | | | | | |
|  | **Rx not filled** | **Rx filled** | **Total Rx** | **Rx Filled %** | **Rx Abandonment %** |
| **Not Readmitted** | 724 | 1139 | 1863 | 61.1% | 38.9% |
| **Readmitted to ER** | 60 | 119 | 179 | 66.5% | 33.5% |
| **Readmitted to ER and IP** | 145 | 204 | 349 | 58.5 | 41.5% |
| **Total** | 929 | 1462 | 2391 | 61.1% | 38.9% |
| Note: Abandonment in both readmitted groups: 38.8% | | | | | |

Supporting Information 1 Table Legend: This appendix represents total Rx fill counts, Rx fill %, and Rx Abandonment % broken out by all maintenance medications and select drug subclasses for the study cohorts.
