# Supplementary material for: Interaction with SMS text-reminders correlate with improved medication adherence and readmission rates for congestive heart failure patients: A retrospective cohort study
Source: PLOS Digit Health. 2025 Dec 31;4(12):e0001157. doi: 10.1371/journal.pdig.0001157 (PMC12755802; doi:10.1371/journal.pdig.0001157)
Supplement: S2 Table — (DOCX) [file pdig.0001157.s002.docx]

### Supporting Information 2 Table. Included Drug Classes for Treatment of Congestive Heart Failure

| S2 Table. Primary CHF Therapeutic Drug Classes |
| --- |
| **Therapeutic Primary Drug Class Names** |
| ACE Inhibitor and Calcium Channel Blocker Combinations |
| ACE Inhibitor and Diuretic Combinations |
| ACE Inhibitors |
| Aldosterone Receptor Antagonists |
| Aldosterone Receptor Antagonists\|Diuretic - Aldosterone Receptor Antagonist, Non-selective |
| Aldosterone Receptor Antagonists\|Diuretic - Aldosterone Receptor Antagonist, Selective |
| Alpha-Beta Blockers |
| Alternative Therapy - Diuretics |
| Angiotensin II Receptor Blocker (ARB)-Beta-Adrenergic Blocker Comb. |
| Angiotensin II Receptor Blocker (ARB)-Calcium Channel Blocker Comb. |
| Angiotensin II Receptor Blocker (ARB)-Calcium Channel Blocker-Diuretic |
| Angiotensin II Receptor Blocker (ARB)-Diuretic Combinations |
| Angiotensin II Receptor Blocker-Neprilysin Inhibitor Comb. (ARNi) |
| Angiotensin II Receptor Blockers (ARBs) |
| Antianginal - Coronary Vasodilators (Nitrates) |
| Antianginal and Anti-ischemic Agents |
| Antianginal and Anti-ischemic Agents, Non-hemodynamic |
| Anticoagulants - Coumarin |
| Anticoagulants - Indanedione |
| Beta Blockers Cardiac Selective |
| Beta Blockers Cardiac Selective, Intrinsic Sympathomimetic Activity |
| Beta Blockers Non-Cardiac Select., Intrinsic Sympathomimetic Activity |
| Beta Blockers Non-Cardiac Selective |
| Calcium Channel Blocker - NSAID, COX-2 Selective Inhibitor Combination |
| Calcium Channel Blockers - Benzothiazepines |
| Calcium Channel Blockers - Dihydropyridines |
| Calcium Channel Blockers - Dihydropyridines - Cerebrovascular Specific |
| Calcium Channel Blockers - Phenylakylamines |
| Cardiac Inotropes |
| Cardiac Inotropes - Phosphodiesterase Inhibitors |
| Cardiac Selective Beta Blocker-Thiazide Diuretic and Related Comb. |
| Cardiovascular Sympathomimetic - Beta-Adrenergic Agonists |
| Cardiovascular Sympathomimetics |
| Cardiovascular Sympathomimetics\|Systemic Sympathomimetic Decongestants |
| Central Alpha-2 Agonists-Thiazide Diuretic and Related Comb. |
| Central Alpha-2 Receptor Agonists |
| Digitalis Glycosides |
| Direct Acting Vasodilators |
| Direct Factor Xa Inhibitors |
| Diuretic - Aldosterone Receptor Antagonist, Non-selective |
| Diuretic - Aldosterone Receptor Antagonist, Selective |
| Diuretic - Arginine Vasopressin V1a/V2 Receptor Antagonists |
| Diuretic - Carbonic Anhydrase Inhibitors |
| Diuretic - Inorganic Salt\|Urinary Acidifier - Others |
| Diuretic - Loop |
| Diuretic - Miscellaneous |
| Diuretic - Osmotic |
| Diuretic - Potassium Sparing |
| Diuretic - Potassium Sparing-Thiazide and Related Combinations |
| Diuretic - Selective Arginine Vasopressin V2 Receptor Antagonists |
| Diuretic - Thiazides and Related |
| Hypertrophic Cardiomyopathy Treatment Agents, Ablative |
| Indirect Factor Xa Inhibitors |
| Non-Cardiac Selective Beta Blocker-Thiazide Diuretic and Related Comb. |
| Platelet Aggregation Inhib - Cyclopentyl-triazolo-pyrimidines (CPTPs) |
| Platelet Aggregation Inhib - PDEsterase &Adenosine deaminase Inhibitor |
| Platelet Aggregation Inhib-PDEsterase and Adenosine deaminase Inhibitr |
| Platelet Aggregation Inhib-Protease-Activ.Receptor-1(PAR-1) Antagonist |
| Platelet Aggregation Inhibitor Combinations |
| Platelet Aggregation Inhibitors - Glycoprotein IIb/IIIa Receptor Inhib |
| Platelet Aggregation Inhibitors - Monoclonal Agents |
| Platelet Aggregation Inhibitors - Phosphodiesterase III Inhibitors |
| Platelet Aggregation Inhibitors - Quinazoline Agents |
| Platelet Aggregation Inhibitors - Salicylates\|Salicylate Analgesics |
| Platelet Aggregation Inhibitors - Salicylates\|Salicylate Analgesics, Buffered |
| Platelet Aggregation Inhibitors - Thienopyridine Agents |
| Platelet Aggregation Inhibitors-Salicylates and Proton Pump Inhib Comb |
| Renin Inhibitor, Direct |
| Renin Inhibitor, Direct and Calcium Channel Blocker Combinations |
| Renin Inhibitor, Direct and Diuretic Combinations |
| Renin-Angiotensin-Aldosterone System (RAAS) Hormones |

Supporting Information 2 Table Legend: This appendix the included drug classes for treatment of Congestive Heart Failure.
